# The AirSeal® insufflation device can entrain room air during routine operation

**DOI:** 10.1007/s10151-020-02291-w

**Published:** 2020-07-30

**Authors:** R. P. Weenink, M. Kloosterman, R. Hompes, P. J. Zondervan, H. P. Beerlage, P. J. Tanis, R. A. van Hulst

**Affiliations:** 1 Department of Anesthesiology, Amsterdam UMC, University of Amsterdam, Amsterdam, The Netherlands; 2 Faculty of Science and Technology, University of Twente, Enschede, The Netherlands; 3 Department of Surgery, Amsterdam UMC, University of Amsterdam, Meibergdreef 9, Amsterdam, The Netherlands; 4 Department of Urology, Amsterdam UMC, University of Amsterdam, Amsterdam, The Netherlands; 5 Department of Hyperbaric Medicine and Department of Anesthesiology, Amsterdam UMC, University of Amsterdam, Amsterdam, The Netherlands

**Keywords:** Laparoscopy, Air embolism, Transanal endoscopic surgery, Insufflation

## Abstract

**Background:**

Surgical procedures that use insufflation carry a risk of gas embolism, which is considered relatively harmless because of the high solubility of carbon dioxide. However, an in vitro study suggested that valveless insufflation devices may entrain non-medical room air into the surgical cavity. Our aim was to verify if this occurs in actual surgical procedures.

**Methods:**

The oxygen percentage in the pneumoperitoneum or pneumorectum/pneumopelvis of eight patients operated with use of the AirSeal® was continuously measured, to determine the percentage of air in the total volume of the surgical cavity.

**Results:**

Basal air percentage in the surgical cavity was 0–5%. During suctioning from the operative field air percentage increased to 45–65%.

**Conclusions:**

The AirSeal® valveless insufflation device maintains optimal distension of the surgical cavity not only by insufflating carbon dioxide, but also by entraining room air, especially during suctioning from the operative field. This may theoretically lead to air embolism in patients operated on with this device.

## Introduction

Venous gas embolism (GE) can occur as a complication of surgical procedures that utilize insufflation. The reported incidence varies widely, and probably depends on the type of procedure and the method used to detect embolization. When surgeons are retrospectively asked to recall clinically significant GE during transanal total mesorectal excision (TaTME), incidence is 0.45% [1]. On the other hand, transesophageal echocardiography detects gas bubbles in up to 100% of laparoscopic hysterectomies [2]. Irrespective of the incidence of intraoperative GE, these bubbles are believed to be subclinical, unless they amount to such a volume that they cause massive pulmonary GE or cardiac air lock. This is based on the assumption that the bubbles consist of carbon dioxide (CO_2_), which, due to its high solubility, will resorb before irreversible damage has occurred.

Recently, Huntington and colleagues published an in vitro study demonstrating that valveless insufflation devices may entrain room air into the surgical cavity [3]. This would put patients at risk of air embolism, which may lead to more severe symptoms because of slower speed of resorption. In this study, we investigated whether the findings of this in vitro study would be replicable during actual surgical procedures.

## Materials and methods

Eight patients who underwent surgery using the AirSeal® (ConMed, Utica, New York, United States) were included. After induction of general anesthesia, a pneumoperitoneum or pneumorectum/pneumopelvis was created. In cases that only involved a pneumoperitoneum, the AirSeal® insufflation tubing was attached to an AirSeal® 8 mm access port placed through the abdominal wall. In combined transanal and abdominal procedures, the abdominal part of the procedure was initiated with a standard insufflation device. For the transanal part of the procedure, the insufflation tubing of the AirSeal® was attached to an AirSeal® 8 mm access port inserted through the gelcap of a GelPOINT path trananal access platform (Applied Medical Resources Corporation, Rancho Santa Margarita, California, United States). An insufflation pressure of 12 mmHg was used uniformly.

**Fig. 1:**
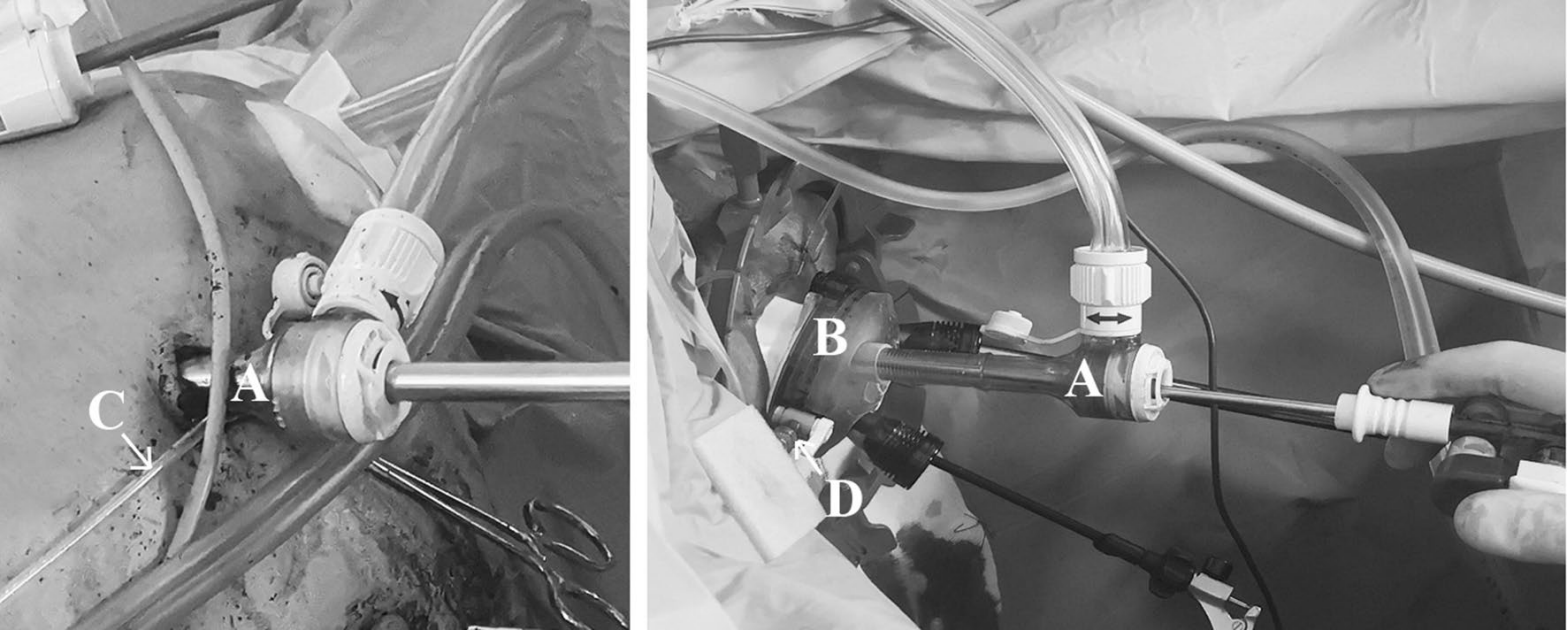
Experimental setup during laparoscopic surgery (left) and transanal surgery (right). (A) AirSeal® 8 mm access port with insufflation tubing attached. (B) GelPOINT path trananal access platform. (C) Sampling line, passed next to the access port into the pneumoperitoneum. (D) luer lock connection on the access platform to which the sampling line is attached (the sampling line itself cannot be seen)

During use of the AirSeal®, a sampling line with an inner diameter of 1.3 mm was placed 10–20 cm into the surgical cavity, either by passing it next to one of the trocars, or through the gel cap of the access platform. Absence of leakage of gas alongside the sampling line was confirmed. If the presence of the sampling line obstructed the surgeon’s view of the operating field, it was attached to one of the luer lock valves of the access platform (in transanal surgery) or the luer lock connector on one of the trocars (in laparoscopic surgery) (Fig. 1).

The sampling line was attached to a calibrated gas analyzer (Fluke Corporation, Everett, Washington, United States). The difference between the pressure in the surgical cavity and atmospheric pressure created a small flow from the surgical field to the gas analyzer, which continuously measured the oxygen concentration in the sample. During the procedure, surgical manipulation such as insertion or removal of an instrument, or suctioning from the operative field was noted. Data analysis was done in MATLAB R2019b (MathWorks, Natick, Massachusetts, United States). The amount of room air as a percentage of the total volume of gas in the surgical cavity at any point in time was calculated as the oxygen percentage multiplied by 4.78, based on the assumptions that (1) room air contains a constant 20.9% of oxygen (100/20.9 = 4.78); (2) room air was the only source of oxygen in the surgical cavity; (3) the gas in the surgical cavity was of homogenous composition; and therefore (4) the gas sample as collected by the analyzer was representative of the contents of the surgical cavity.

## Results

**Fig. 2:**
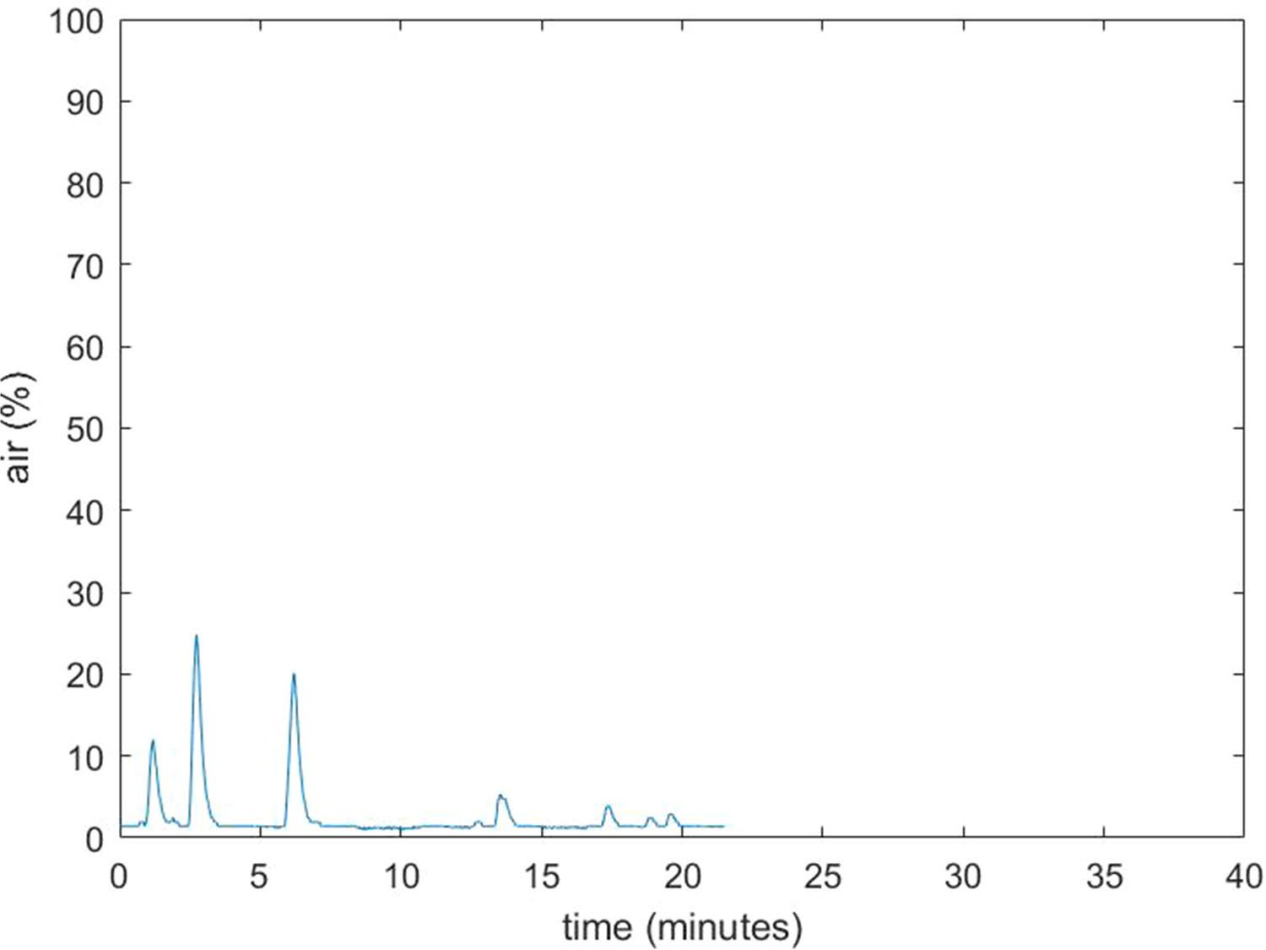
Example of air percentage in the pneumorectum of case 4 during normal use (no suctioning)

**Fig. 3:**
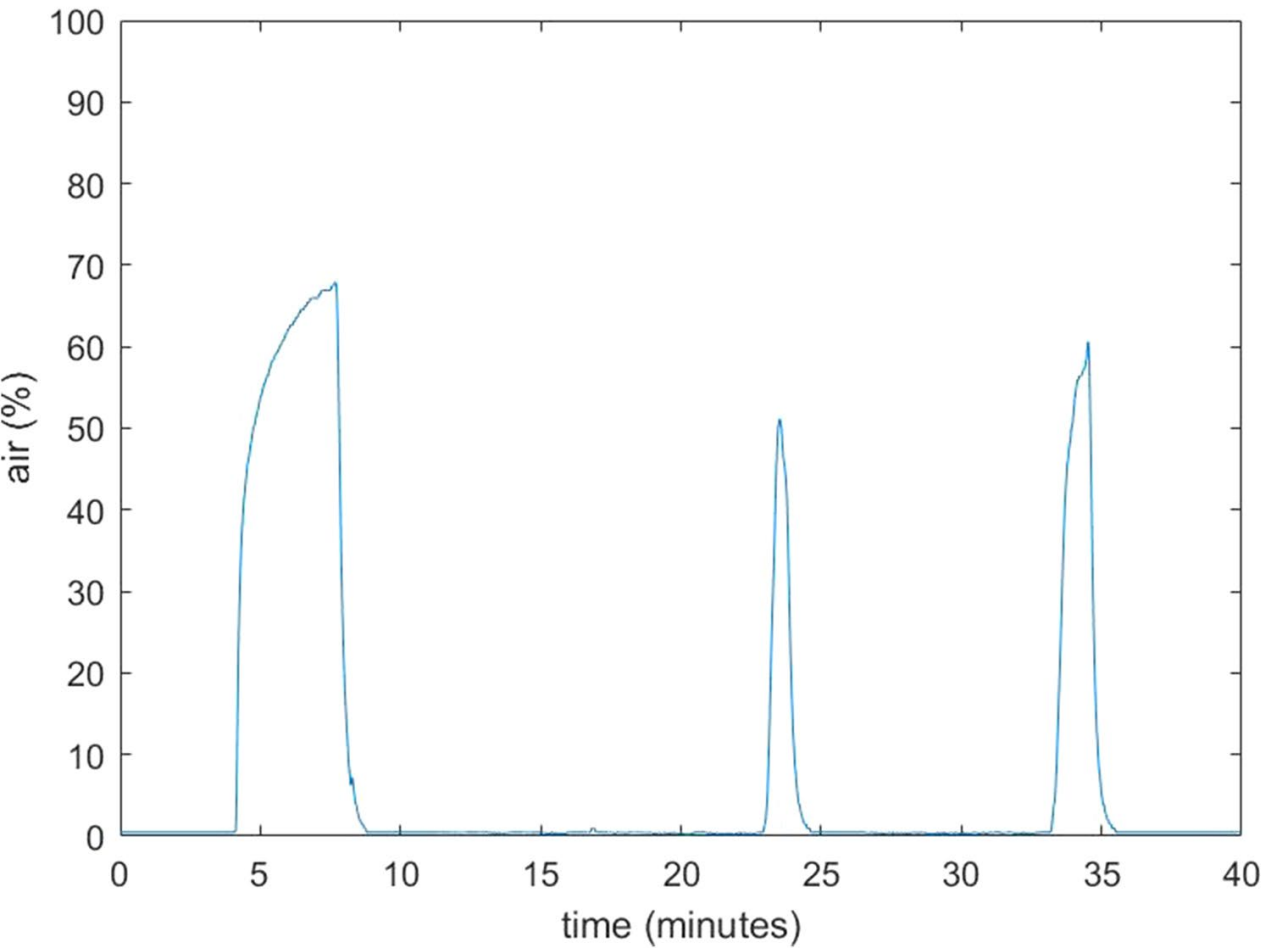
Example of air percentage in the pneumorectum of case 7. The three peaks in air percentage correspond to short periods of suctioning from the surgical field

**Fig. 4:**
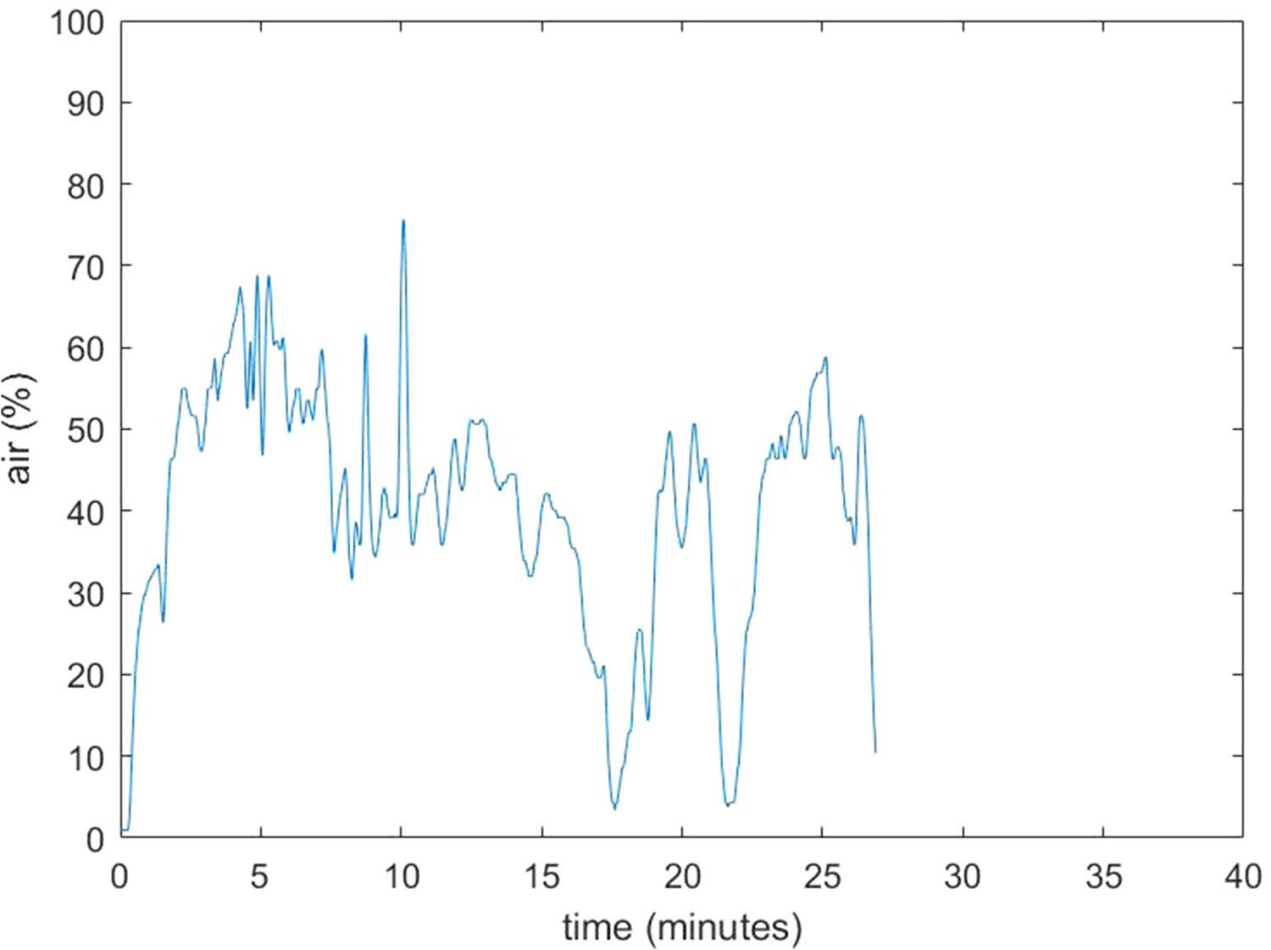
Example of air percentage in the pneumopelvis of case 3. During this phase of the operation, there were many periods of suctioning from the surgical field due to diffuse venous bleeding

Details of the eight patients included in this study are provided in Table 1. All surgical procedures were uneventful; specifically, no signs of GE were observed. During normal surgical conditions, the amount of room air as a percentage of total volume of the surgical cavity was 0–5%. However, multiple increases in air percentage were seen in all procedures. Some of these increases could be related to removal and reinsertion of a surgical instrument through the laparoscopic ports. This lead to relatively small increases of air, up to 10–25%, which immediately restored to baseline values (Fig. 2). When suctioning with 40 l/min through a standard 5 mm laparoscopic aspirator was performed, the air percentage increased to higher values, around 45–65% (Figs. 3 and 4). Maximum observed flow rate of CO_2_ as displayed on the AirSeal® during suctioning was 9 l/min, while at the same time no decrease in the distention of the surgical cavity was notable. During suctioning, when the AirSeal® access port was not occupied with a surgical instrument, flow of air from the operating room into the surgical cavity could be felt by placing a finger on the access port. Placement of the supplied noise cancelling cap on the access port did not prevent air entrainment. We observed no differences in measured values, whether the sampling line was placed in the surgical cavity, or attached to a luer lock connector on a trocar or on the access platform.

**Table 1:** Details of the surgical procedures performed in 8 patients

Patient	Sex	Age (years)	Indication	Procedure	Use of AirSeal
1	M	65	Renal cell carcinoma	Nephrectomy and adrenalectomy	Pneumoperitoneum
2	M	19	Ulcerative colitis, status post subtotal colectomy	Completion proctectomy with ileoanal pouch	Pneumoperitoneum and pneumorectum/-pelvis
3	V	74	Rectal fistula post rupture of sphincter with dynamic graciloplasty and sacrocolpopexy	Proctectomy, removal of mesh and pelvic omentoplasty	Pneumoperitoneum and pneumorectum/-pelvis
4	M	43	Ulcerative colitis, status post subtotal colectomy	Completion proctectomy with ileoanal pouch	Pneumoperitoneum and pneumorectum/-pelvis
5	M	78	Presacral sinus after low anterior resection	Intersphincteric resection of colorectal anastomosis and pelvic omentoplasty	Pneumoperitoneum and pneumorectum/-pelvis
6	M	49	Ulcerative colitis, status post subtotal colectomy	Completion proctectomy with ileoanal pouch	Pneumoperitoneum and pneumorectum/-pelvis
7	F	72	Rectal cancer	Transanal total mesorectal excision	Pneumoperitoneum and pneumorectum/-pelvis
8	F	35	Space-occupying lesion	Partial nephrectomy	Pneumoperitoneum

## Discussion

Our data confirms the observation made in an in vitro study [3] that the valveless AirSeal® insufflation device can entrain room air into the surgical cavity. This occurs especially during suctioning from the operative field, but also with removal and insertion of instruments. Airflow into the surgical cavity could indeed be felt at the AirSeal® trocar during suctioning. The maximum air percentage of 65% corresponds with the value found in the in vitro study, when the same suctioning flow of 40 l/min was used [3].

CO_2_ is the laparoscopic gas of choice because it is readily available, inert, non-combustible, and has a high solubility in blood. This latter property renders CO_2_ relatively safe in cases of intravascular introduction of gas. Air is 20 times less soluble in blood than CO_2_, mainly due to the low solubility of the nitrogen it contains, which makes air emboli potentially much more harmful [4]. Indeed, studies suggest that abrupt introduction of 200 ml of air into the venous vasculature of an adult is lethal, while this amount is approximately 1000 ml for CO_2_ [4]. Other disadvantages of using air as insufflation gas are that it results in more postoperative shoulder pain, caused by intraperitoneal air retained for a prolonged duration, as well as an increased risk of combustion [4]. Additionally, when room air instead of medical grade air is used, the introduction of contaminants from the operating room cannot be excluded.

Suctioning from the operative field is often performed in cases of bleeding, which is also a risk factor for gas embolization [5]. Potentially even more dangerous is direct vascular injury that is not accompanied by bleeding. This situation may occur during laparoscopic or endoscopic surgery in which the operating field is above the level of the heart, which results in venous pressure being lower than insufflation pressure, such as TaTME. Under these circumstances, vascular injury may lead to massive flow of insufflation gas into the vasculature [5]. It is highly likely that a valveless device such as the AirSeal® will respond to the ensuing loss of pressure in the same way that is does when pressure is lost due to suctioning, namely by sucking air into the surgical cavity. This would then lead to influx of not only CO_2_ [6], but potentially also of air. One could hypothesize that this mechanism may explain the relatively high incidence of clinically significant GE in TaTME [1].

Our study has a few drawbacks, which however do not weaken the validity of our findings. The small number of patients may limit the generalizability of our results. Our findings, however, were observed in all 8 patients, and we have no reason to assume that inclusion of more patients or other types of procedures would lead to different conclusions. We have tested only the AirSeal® device, so care should be taken when extrapolating our findings to other insufflation devices. Specifically, we did not compare our results to findings in traditional (non-valveless) devices, but a study in 14 laparoscopies using a non-valveless device showed a mean air percentage of only 3.2% [7]. In this study, which was published as an abstract, average maximum amount of oxygen percentage in 16 patients in whom the AirSeal® was used was 43%, which corresponds with our findings. Lastly, our study was not designed to actually determine the occurrence of GE. Because of the low incidence of clinically significant GE this would require a much larger study, ideally with retrieval of gas bubbles from the vessels to determine their composition.

## Conclusions

The AirSeal® device maintains optimal insufflation not only by insufflating CO_2_, but also by entraining room air, particularly during sudden decrease of pressure as occurs during suctioning. We believe the surgical and anesthesiological community should be aware of this phenomenon, most importantly because gas emboli occurring during use of this device cannot be assumed to be ‘harmless’ CO_2_ emboli, but may also contain air. We urge manufacturers of insufflation devices to optimize their design in order to prevent entrainment of air.

## Data Availability

All original data is available from the authors.
